# Emodin potentiates the antiproliferative effect of interferon α/β by activation of JAK/STAT pathway signaling through inhibition of the 26S proteasome

**DOI:** 10.18632/oncotarget.6616

**Published:** 2015-12-14

**Authors:** Yujiao He, Junmei Huang, Ping Wang, Xiaofei Shen, Sheng Li, Lijuan Yang, Wanli Liu, Apichart Suksamrarn, Guolin Zhang, Fei Wang

**Affiliations:** ^1^ Key Laboratory of Natural Medicine and Clinical Translation, Chengdu Institute of Biology, Chinese Academy of Sciences, Chengdu, China; ^2^ School of Chinese Pharmacy, Chengdu University of Traditional Chinese Medicine, Chengdu, China; ^3^ Department of Chemistry and Center for Innovation in Chemistry, Faculty of Science, Ramkhamhaeng University, Bangkok, Thailand; ^4^ MOE Key Laboratory of Protein Science, School of Life Sciences, Tsinghua University, Beijing, China; ^5^ Sichuan Translational Medicine Research Hospital, Chinese Academy of Sciences, Chengdu, China

**Keywords:** emodin, interferon, JAK/STAT, 26S proteasome

## Abstract

The 26S proteasome is a negative regulator of type I interferon (IFN-α/β) signaling. Inhibition of the 26S proteasome by small molecules may be a new strategy to enhance the efficacy of type I IFNs and reduce their side effects. Using cell-based screening assay for new 26S proteasome inhibitors, we found that emodin, a natural anthraquinone, was a potent inhibitor of the human 26S proteasome. Emodin preferably inhibited the caspase-like and chymotrypsin-like activities of the human 26S proteasome and increased the ubiquitination of endogenous proteins in cells. Computational modeling showed that emodin exhibited an orientation/conformation favorable to nucleophilic attack in the active pocket of the β1, β2, and β5 subunits of the 26S proteasome. Emodin increased phosphorylation of STAT1, decreased phosphorylation of STAT3 and increased endogenous gene expression stimulated by IFN-α. Emodin inhibited IFN-α-stimulated ubiquitination and degradation of type I interferon receptor 1 (IFNAR1). Emodin also sensitized the antiproliferative effect of IFN-α in HeLa cervical carcinoma cells and reduced tumor growth in Huh7 hepatocellular carcinoma-bearing mice. These results suggest that emodin potentiates the antiproliferative effect of IFN-α by activation of JAK/STAT pathway signaling through inhibition of 26S proteasome-stimulated IFNAR1 degradation. Therefore, emodin warrants further investigation as a new means to enhance the efficacy of IFN-α/β.

## INTRODUCTION

Type I interferons (IFN-α and IFN-β) play central roles in the innate immune response and exhibit antiviral, antiproliferative, and immunomodulatory effects by activation of Janus kinase/signal transducer and activator of transcription (JAK/STAT) pathway signaling [1]. Clinically, type I IFNs are widely used in the treatment of viral diseases such as hepatitis B and hepatitis C, as well as tumors such as hepatocellular carcinoma and leukemia. However, the efficacy and side effects of type I IFNs are correlated with their dosage and duration of use [2]. Therefore, small-molecule activators of JAK/STAT signaling, which could amplify the effects of type I IFNs, are high-priority targets of drug development efforts. Intriguingly, several small-molecular activators of type I IFNs have been identified through cell-based screening and exhibit antiviral or anti-cancerous effects through various mechanisms, including suppression of cAMP-PKA-SHP2 signaling, inhibition of pyrimidine biosynthesis, and activation of type I IFN receptors [3–5]. JAK/STAT signaling pathway stimulated by type I IFNs is regulated in a complex and tissue-specific manner; therefore, new activators of JAK/STAT pathway will facilitate the development of new strategies for IFN therapy [6].

The 26S proteasome, a molecular complex that catalyzes the degradation of ubiquitinated proteins, participates in negative regulation of JAK/STAT pathway signaling. Binding of type I IFNs with IFNAR1/2 induces ubiquitination, endocytosis, and lysosomal degradation of the IFNAR1 and is an important pathway through which IFN signaling is attenuated [7]. In addition, IFNAR1 is stabilized by its binding to Tyk2 kinase [8]. Activated STAT1 is degraded in the 26S proteasome by a mechanism involving the F-box E3 ligase SCF^βTrcp^ [9]. Simian virus 5 inhibits IFN signaling by specifically targeting STAT1 for proteasomal degradation [10]. The negative regulatory effects of IFN-β on osteoclastogenesis are correlated with the expression level of Jak1, which is regulated by receptor activator of nuclear factor κB ligand (RANKL) by inducing it for ubiquitination and proteasomal degradation [11]. Therefore, inhibition of the 26S proteasome activity is a strategy that could be utilized to suppress its attenuating effect on IFN signaling and thus enhance the efficacy of IFN, and methods of producing such inhibition merit further study.

Emodin (1,3,8-trihydroxy-6-methylanthraquinone) is a naturally occurring anthraquinone derivative isolated from the roots and bark of numerous plants, as well as molds and lichens. Emodin is an active constituent of several herbs used in traditional Chinese medicine, including *Rheum palmatum* and *Polygonam multiflorum*, and has diuretic, vasorelaxant, anti-bacterial, anti-viral, anti-ulcerogenic, anti-inflammatory, and anti-cancer effects [12]. Because of the promising chemopreventive and chemotherapeutic potential of emodin, extensive efforts have been aimed at exploiting its mechanism and many reports suggest that emodin efficiently suppresses multiple cell signaling pathways, including p53, NF-κB, and AKT/mTOR signaling [13]. Despite evidence that emodin directly interacts with several molecular targets involved in inflammation and cancer, including casein kinase II, Her2/neu, topoisomerase II, and heat shock protein 90 (Hsp90) [13], it is unclear whether other proteins are involved in the mechanism by which emodin exerts its pharmacological effects; however, a comprehensive understanding of its mechanism of action is important for the development of emodin as a new therapeutic agent.

Emodin exhibits anticancer effects partly through regulation of JAK/STAT pathway signaling. Emodin suppresses activation of JAK/STAT signaling in leukemia cells by inhibiting the kinase CK2, inhibits interleukin-6-induced JAK2/STAT3 signaling in myeloma cells, and suppresses STAT3 activation through upregulation of SHP-1 in hepatocellular carcinoma cells [14–16]. Previously, we showed that emodin is an activator of type I IFN-induced JAK/STAT signaling and could increase expression of endogenous antiviral genes induced by type I IFNs [17]. However, the mechanism by which emodin acts on type I IFN-induced JAK/STAT signaling is unclear. In this study, we determined whether emodin is a potent inhibitor of 26S proteasome that activates type I IFN-induced JAK/STAT signaling through inhibition of 26S proteasome-stimulated IFNAR1 degradation.

## RESULTS

### Establishment of an ubiquitin-independent cell-based assay for 26S proteasome inhibitors

**Figure 1:**
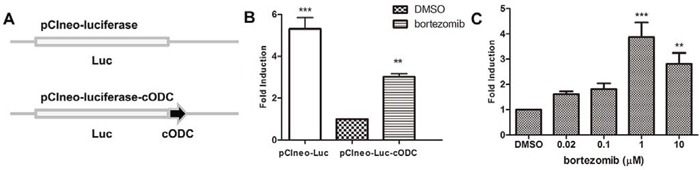
Establishment of an ubiquitin-independent cell-based assay for 26S proteasome inhibitors **A.** Schematic diagram of the luciferase reporter with or without the cODC motif. cODC is an ubiquitin-independent domain of ornithine decarboxylase that is required for 26S proteasome degradation. **B.** HEK293A cells were co-transfected in a 24-well plate with 0.1 μg of the pCIneo-luciferase or pCIneo-luciferase-cODC plasmids with 0.3 μg pSV-β-galactosidase expression plasmid. After 24 h of incubation, the cells were lysed and luciferase activity was measured and normalized to β-galactosidase activity. The results are expressed as relative-fold induction, referring to the ratio of normalized luciferase activity measured in the cells relative to the activity observed in the pCIneo-luciferase-cODC-transfected cells. **C.** HEK293A-luciferase-cODC cells were seeded in a 96-well plate and treated with various concentrations of bortezomib for 6 h. The cells were lysed and luciferase activity was measured. The results are expressed as relative-fold induction, referring to the ratio of normalized luciferase activity measured in bortezomib-treated cells relative to the activity observed in DMSO-treated cells.

Native ornithine decarboxylase (ODC) is recognized for degradation by the 26S proteasome without ubiquitin modification [18]. Vertebrate forms of ODC have a small conserved degradation tag (37 amino acids in mice and humans) at the C terminus (cODC), where strictly unidirectional proteasomal degradation begins [19]. Fusing this degradation tag to other proteins, including those of mammals, plants, and fungi, promotes their rapid turnover by the eukaryotic proteasome [20]. To monitor inhibition of the 26S proteasome, the pCIneo-luciferase-cODC plasmid was constructed. As illustrated in Figure 1A, the mouse cODC was fused to the C terminus of the luciferase reporter to promote proteasomal degradation of luciferase. The pCIneo-luciferase plasmid lacking the cODC tag was used as a negative control plasmid to exclude the effect of non-specific luciferase reporter activity inhibited by the test compounds. To examine whether the cODC tag promotes proteasomal degradation of luciferase, HEK293A cells were transiently transfected with equal amounts of the pCIneo-luciferase and pCIneo-luciferase-cODC plasmids. The activity of luciferase fused with the cODC tag was significantly lower than that of luciferase without the cODC tag. The activity of luciferase-cODC was increased when the cells were treated with the 26S proteasome inhibitor bortezomib (Figure 1B), indicating that luciferase-cODC was specifically degraded by the 26S proteasome. A HEK293A cell line stably transfected with the pCIneo-luciferase-cODC plasmid (HEK293A-luciferase-cODC) was generated and treated with bortezomib. Treatment with bortezomib (at 0.02–10 μM) increased luciferase activity in the HEK293A-luciferase-cODC cells in a concentration-dependent manner (Figure 1C). These results suggest that degradation of the luciferase reporter in HEK293A-luciferase-cODC cells is 26S proteasome-specific, indicating that they can be used to screen new proteasome inhibitors.

### Identification of emodin as an inhibitor of the 26S proteasome

**Figure 2:**
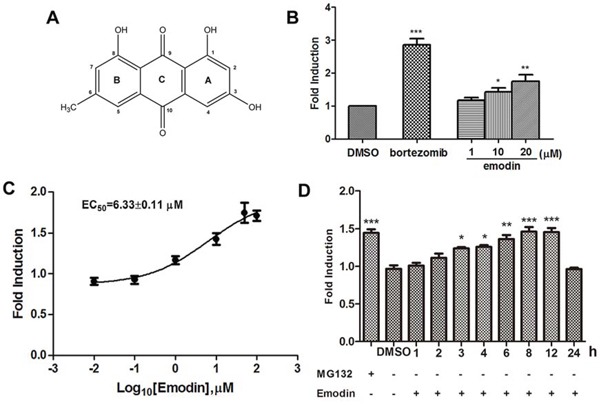
Emodin inhibits the activity of the 26S proteasome in vitro and in vivo **A.** The chemical structure of emodin. **B.** HEK293A-luciferase-cODC cells were seeded in 96-well plates and treated in the presence of the indicated concentrations of emodin and 1 μM bortezomib for 3 h. **C.** The concentration-response curve of emodin. The calculated EC_50_ of emodin was 6.33 μM. **D.** HEK293A-luciferase-cODC cells were seeded in 96-well plates and treated with 10 μM emodin for the indicated period or treated with 10 μM MG132 for 2 h. The results are representative of 3 separate experiments. The error bars represent the standard deviation of the measurements. (*) *p* < 0.05, (**) *p* < 0.01, (***) *p* < 0.001 in comparison with the DMSO control.

Using the HEK293A-luciferase-cODC cell line, we screened a chemical library containing 1431 natural products and synthesized analogues [14]. After hit reconfirmation, emodin was identified to potently increase luciferase reporter expression. The chemical structure of emodin is illustrated in Figure 2A. Emodin at concentrations of 1–20 μM inhibited luciferase-cODC degradation in a concentration-dependent manner (Figure 2B). The EC_50_ value of emodin for inhibition of luciferase-cODC degradation was 6.33 μM (Figure 2C). Emodin did not show cytotoxicity in the HEK293A-luciferase-cODC cells at concentrations of 1–20 μM (data not shown). Promotion of luciferase-cODC expression by 10 μM emodin was first evident after 3 h of exposure and was sustained until the 12 h time point, when luciferase-cODC expression was increased by approximately 1.5-fold in comparison with that of the control cells (Figure 2D). Compared with that, the proteasome inhibitor MG132 at 10 μM more potently promoted luciferase-cODC expression from 2 h until to 24 h (Figure S1). We also examined the effect of emodin on GFP-CL1, a reporter for the proteasome activity in vivo. The result showed that emodin significantly promoted the accumulation of GFP-CL1 in cells, similar as MG132 did (Figure S2). These results indicate that emodin inhibits the 26S proteasome.

### Inhibitory effect of emodin on 26S proteasome activity

**Figure 3:**
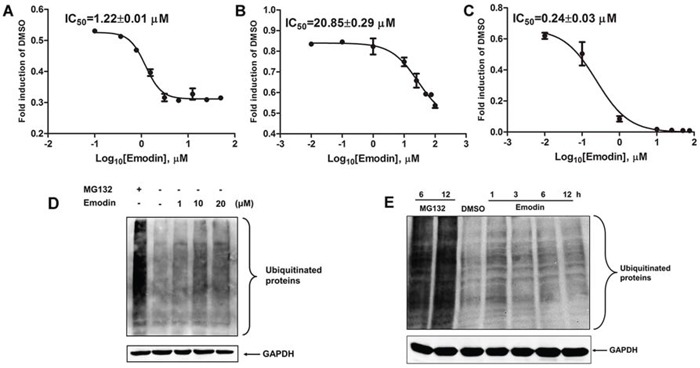
Inhibitory effect of emodin on 26S proteasome activity The purified human 26S proteasome (0.1 μg) was treated with or without different concentrations of emodin and **A.** 40 μM Suc-Leu-Leu-Val-Tyr-AMC (for measurement of chymotrypsin-like activity), **B.** 40 μM Ac-Arg-Leu-Arg-AMC (for measurement of trypsin-like activity), or **C.** 40 μM Z-Nle-Pro-Nle-Asp-aminoluciferin (for measurement of caspase-like activity) for 2 h at 37°C. HeLa cells were treated with the indicated concentrations of emodin or 10 μM MG132 for 3 h **D.**, or with 20 μM emodin or 10 μM MG132 for the indicated durations **E.**, and the cell lysates were probed with anti-ubiquitin antibodies. GAPDH was used as an internal control.

Purified human 26S proteasome was used to examine whether emodin directly inhibited the 26S proteasome. As shown in Figure 3A, emodin inhibited the chymotrypsin-like activity of the 26S proteasome, with an IC_50_ value of 1.22 μM. Emodin also inhibited the trypsin-like and caspase-like activities of the 26S proteasome, with IC_50_ values of 20.85 μM and 0.24 μM, respectively (Figure 3B and 3C). To examine the effect of emodin on endogenous protein ubiquitination, HEK293A cells were treated with emodin and the cell lysates were probed with anti-ubiquitin antibodies. As shown in Figure 3D, proteasome inhibitor MG132 significantly increased ubiquitinated protein accumulation in comparison with that of the untreated cells. In addition, emodin also increased endogenous protein ubiquitination in a concentration-dependent manner. Furthermore, emodin treatment also significantly increased endogenous protein ubiquitination in a time-dependent manner (Figure 3E). These results indicate that emodin is a potent inhibitor of the 26S proteasome.

### Molecular docking of emodin with proteasome subunits

**Figure 4:**
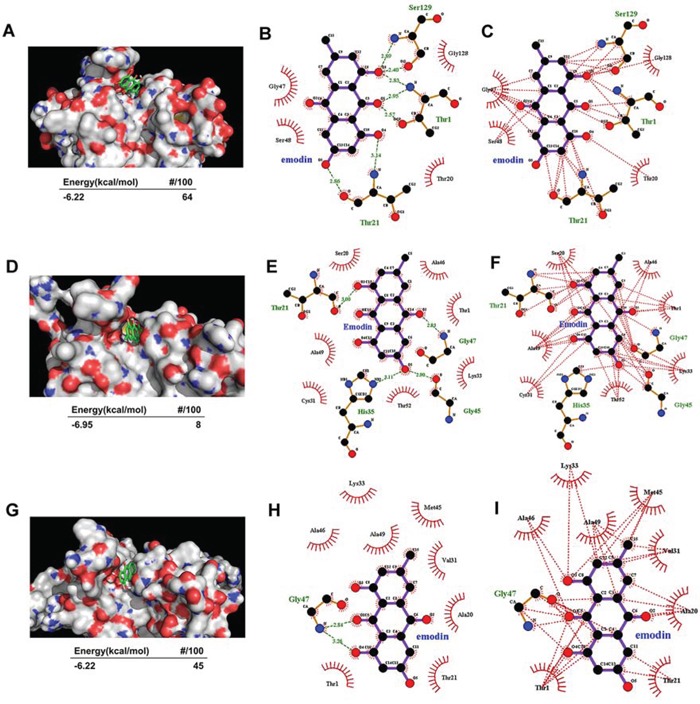
Molecular docking of emodin on the proteasome subunits **A–C.** Emodin was docked to the β5 subunit of the proteasome. The energy of the inhibitory conformation and the number of runs (out of 100) that adopted the inhibitory conformation are shown below A. The threonine catalytic residue and all amino acids in the S1 pocket of the β5 subunit involved in the formation of H-bonds B. and hydrophobic interactions C. with emodin are highlighted. **D–F.** Emodin was docked to the β2 subunit of the proteasome. The energy of the inhibitory conformation and the number of runs (out of 100) that adopted the inhibitory conformation are shown below D. The threonine catalytic residue and all amino acids in the S1 pocket of the β2 subunit involved in the formation of H-bonds E. and hydrophobic interactions F. with emodin are highlighted. **G–I.** Emodin was docked to the β1 subunit of the proteasome. The energy of the inhibitory conformation and the number of runs (out of 100) that adopted the inhibitory conformation are shown below G. The threonine catalytic residue and all amino acids in the S1 pocket of the β1 subunit involved in the formation of H-bonds H. and hydrophobic interactions I. with emodin are highlighted.

The proteolytic activities of the proteasome are dependent on the N-terminal threonine (Thr^1^) residue hydroxyl group of the β subunits, which are responsible for catalyzing the cleavage of peptides through nucleophilic attack. An *in silico* docking study was performed to aid the understanding of possible binding modes of emodin with the active pocket of proteasome subunits and subsequent proteasome inhibition. Emodin was docked to the active site of the proteasome β1, β2, and β5 subunits, which are responsible for caspase-like, trypsin-like, and chymotrypsin-like activities of the proteasome, respectively. As shown in Figure 4A, emodin adopted a conformation favorable for nucleophilic attack at the active site of the β1 subunit with energy of −6.22 kcal/mol. To identify favorable binding modes of emodin to the proteasomal chymotrypsin-like active site, we analyzed hydrogen-bond (H-bond) formation and hydrophobic interactions between emodin and the β1 subunit. There are 3 polar hydrogens and 1 carbonyl-oxygen in emodin that are available for H-bonding and participate in H-bonding with the Thr^1^, Thr^21^, and Ser^129^ residues of the β1 subunit (Figure 4B). Emodin was also favorably inserted within the S1 hydrophobic pocket of the β1 subunit through hydrophobic interactions (Figure 4C). Similarly, emodin adopted a conformation favorable for nucleophilic attack at the active site of the β2 subunit with energy of −6.95 kcal/mol (Figure 4D). There are 2 polar hydrogens and 1 carbonyl-oxygen on emodin that are available for H-bonding and which participate in H-bonding with the Thr^21^, His^35^, Gly^45^, and Gly^47^ residues of the β2 subunit (Figure 4E). Emodin was favorably inserted within the S1 hydrophobic pocket of the β2 subunit through hydrophobic interactions (Figure 4F). Emodin adopted a conformation favorable for nuleophilic attack at the active site of the β5 subunit with energy of −6.22 kcal/mol (Figure 4G). There is 1 polar hydrogen on emodin that is available for H-bonding, which participates in H-bonding with the Gly^47^ residue of the β5 subunit (Figure 4H). Emodin was favorably inserted within the S1 hydrophobic pocket of the β2 subunit through hydrophobic interactions (Figure 4I). These results suggest that emodin can exhibit an orientation/conformation in proximity to the N-terminal Thr^1^ of the β1, β2, and β5 subunits and is thus subject to nucleophilic attack.

### Emodin promotes IFN-α/β-induced activation of JAK/STAT signaling

**Figure 5:**
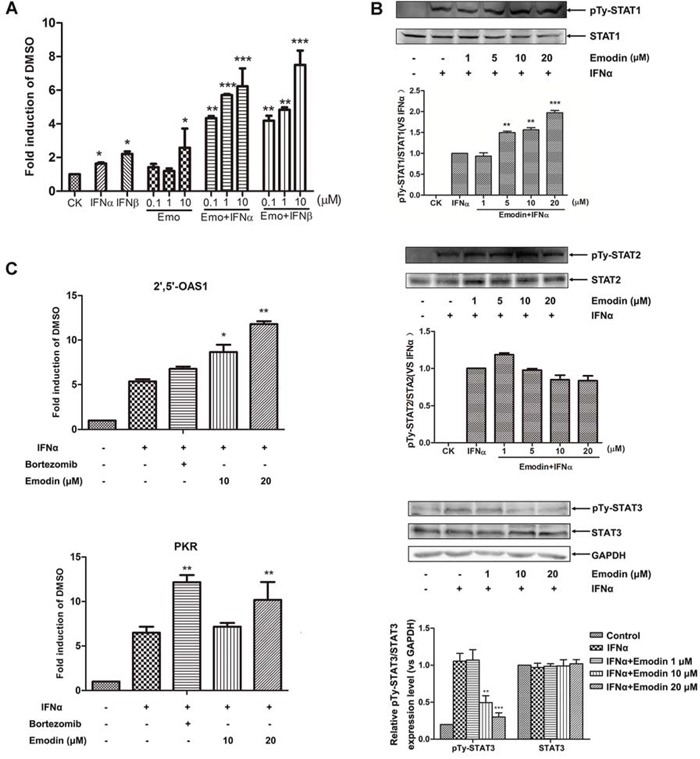
Emodin enhances IFN-α/β-induced JAK/STAT pathway activation **A.** The HepG2-ISRE-Luc2 cells were seeded in 96-well plates (1 × 10^4^/well) and treated with various concentrations of emodin for 2 h, followed by the addition of 200 U/mL IFN-α or IFN-β for 24 h. **B.** HEK293A cells were incubated with the indicated concentrations of emodin for 2 h, after which 200 U/mL IFN-α was added for 1 h. The cell lysates were immunoblotted with antibodies against phospho-STAT1 (Tyr701), STAT1, phospho-STAT2 (Tyr690), STAT2, phosphor-STAT3 (Tyr705) and STAT3 respectively. Quantitative results are depicted. **C.** HEK293A cells were treated 200 U/mL IFN-α with or without 10 μM bortezomib or with the indicated concentrations of emodin for 24 h. Real-time PCR was used to determine the mRNA expression of PKR and 2′5′-OAS1. The results are presented as induction (n-fold) relative to basal levels in untreated cells. GAPDH was used as an internal control. (*) *p* < 0.05, (**) *p* < 0.01, (***) *p* < 0.001 vs. control (*n* = 3). CK, DMSO control.

We showed that emodin alone can promote endogenous IFN-α-stimulated genes expression, but its effect on JAK/STAT signaling is unknown. The 26S proteasome participates in negative regulation of JAK/STAT signaling, so it is possible that emodin may promote the activation of type I IFN-induced JAK/STAT signaling by inhibiting the activity of the 26S proteasome. To test this hypothesis, we first examined the effect of emodin on IFNα/β-induced JAK/STAT signaling. As shown in Figure 5A, emodin potently increased ISRE luciferase reporter expression induced by IFN-α or IFN-β in a concentration-dependent manner. Next, we examined the effect of emodin on phosphorylation of STAT1, STAT2 and STAT3 in combination with IFN-α. In comparison with IFN-α alone, emodin increased tyrosine phosphorylation of STAT1 and decreased tyrosine phosphorylation of STAT3 in a concentration-dependent manner, but had no effect on STAT2 phosphorylation (Figure 5B). PKR and 2′, 5′-OAS1 are IFN-α-responsive genes that contain ISRE consensus sequences in their promoter regions. We examined the effect of emodin in combination with IFN-α on mRNA expression of PKR and 2′, 5′-OAS1. As shown in Figure 5C, mRNA expression levels of both IFN-stimulated genes significantly increased after treatment with a combination of emodin and IFN-α in comparison with levels measured after treatment with IFN-α alone. These results indicate that emodin enhances activation of type I IFN-induced JAK/STAT pathway signaling.

### Emodin inhibits ubiquitination and degradation of IFNAR1

**Figure 6:**
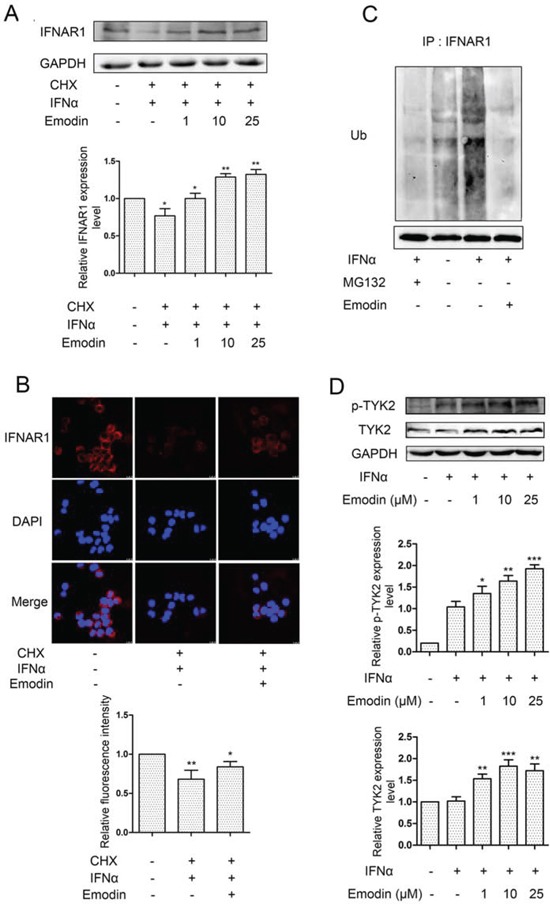
Emodin inhibits IFN-α-induced degradation of IFNAR1 **A.** HEK293A cells were treated with 20 μM cycloheximide (CHX) for 2 h, followed by the addition of emodin (1 μM, 10 μM, or 25 μM) for 12 h and treatment with IFN-α (1 × 10^4^ U/mL) for 2 h. The cell lysates were immunoblotted with anti-IFNAR1 antibodies. GAPDH staining is shown as a loading control. Quantitative results are depicted. **B.** HeLa cells were treated with emodin (10μM) for 12 h and IFN-α (1 × 10^4^ U/mL) for 2 h in the presence of CHX (20 μM) for 2 h. The cells were processed for immunofluorescence using IFNAR1 antibody. **C.** HeLa cells were incubated with MG132 (20 μM) or emodin (20 μM) for 12 h, followed by treatment with IFN-α (1 × 10^4^ U/mL) for 2 h, and the cell lysates were immunoprecipitated with the anti-IFNAR1 antibodies. Immunoblotting was performed using anti-ubiquitin antibodies. Anti-GAPDH antibody staining represents 5% of the total cell lysates used in immunoprecipitation. **D.** HeLa cells grown in 6-well plates were treated with emodin (1 μM, 10 μM, or 25 μM) for 12 h, followed by the addition of 2000 U/mL IFN-α for 30 min. The cells were harvested and processed for western blotting. GAPDH was used as internal control. Quantitative results are depicted. (*) *p* < 0.05, (**) *p* < 0.01, (***) *p* < 0.001 vs. control (*n* = 3).

IFN-α-induced ubiquitination and lysosomal degradation of IFNAR1 are key steps in the negative regulation of IFN signaling. Thus, inhibition of the activity of the 26S proteasome by emodin may suppress IFN-α-induced ubiquitination and degradation of IFNAR1. To test this hypothesis, we examined the effect of emodin on IFNAR1 degradation. As shown in Figure 6A, in the presence of cycloheximide (CHX) (a blocker of *de novo* protein synthesis), IFN-α treatment stimulated IFNAR1 degradation, as demonstrated in a previous report [8]. The addition of emodin significantly inhibited IFNAR1 degradation stimulated by IFN-α. Immunofluorescence staining of IFNAR1 also showed that emodin inhibited IFN-α-stimulated IFNAR1 degradation (Figure 6B). IFNAR1 is ubiquitinated prior to lysosomal degradation, which can be inhibited by 26S proteasome inhibitors [8]. Therefore, we examined the effect of emodin on IFNAR1 ubiquitination. As shown in Figure 6C, the stimulatory effect of IFN-α treatment on IFNAR1 ubiquitination was inhibited by 26S proteasome inhibitor MG132 or emodin. Tyk2 enhances surface IFNAR1 expression and STAT1 activation [8]. We then examined the effect of emodin on Tyk2. Emodin significantly increased the phosphorylation and expression of Tyk2 in the presence of IFN-α in a concentration-dependent manner (Figure 6D). These results indicate that emodin protects IFNAR1 from degradation.

### Emodin promotes the antiproliferative effect of IFN-α

**Figure 7:**
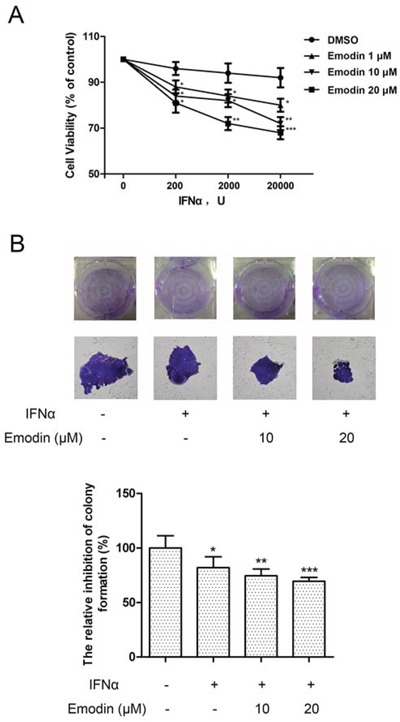
Emodin potentiates the antiproliferative effect of IFN-α (A) The HeLa cells (5 × 10^3^ cells/well) were seeded in 96-well plates and treated with the indicated concentrations of emodin and IFN-α for 72 h. Cell viability was measured using an Alamar Blue assay. The values are expressed as the percentage cell viability relative to the DMSO-treated control cells. (B) The HeLa cells growing in 6-well plates were treated with the indicated concentrations of emodin and IFN-α (1 × 10^4^ U/mL ) for 12 days, and then colonies were visualized by staining with crystal violet and counted manually. The bar graph was obtained by calculating the percentages of colony numbers from each well relative to the DMSO-treated control. (*) *p* < 0.05, (**) *p* < 0.01, (***) *p* < 0.001 vs. control (*n* = 3).

To examine whether emodin enhanced the anti-proliferative effect of IFN-α, human cervical cancer HeLa cells were treated with emodin in combination with IFN-α. As shown in Figure 7A, IFN-α treatment inhibited the proliferation of HeLa cells. The addition of emodin significantly enhanced antiproliferative effect of IFN-α in a dose-dependent manner, whereas emodin at concentrations of 1–20 μM had no effect on HeLa cell proliferation (data not shown). Furthermore, treatment with IFN-α resulted in decreased colony formation in HeLa cells compared with the untreated control cells. The addition of emodin significantly decreased the number of colonies, compared with cells treated by IFN-α alone (Figure 7B). To examine whether activation of JAK/STAT pathway is required for the antiproliferative effect of emodin, we treated the cells with JAK inhibitor and found that it completely abolished the antiproliferative effect of emodin in combination of IFN-α (Figure S3). To test whether the antiproliferative effect of emodin depends on the presence of STAT1, we knocked down the expression of STAT1 by siRNA transfection. The result showed that knockdown of STAT1 partially abolished the antiproliferative effect of emodin in combination of IFN-α (Figure S4). These results suggest that emodin enhances the antiproliferative effect of IFN-α in vitro.

### Emodin promotes the antiproliferative effect of IFN-α *in vivo*

**Figure 8:**
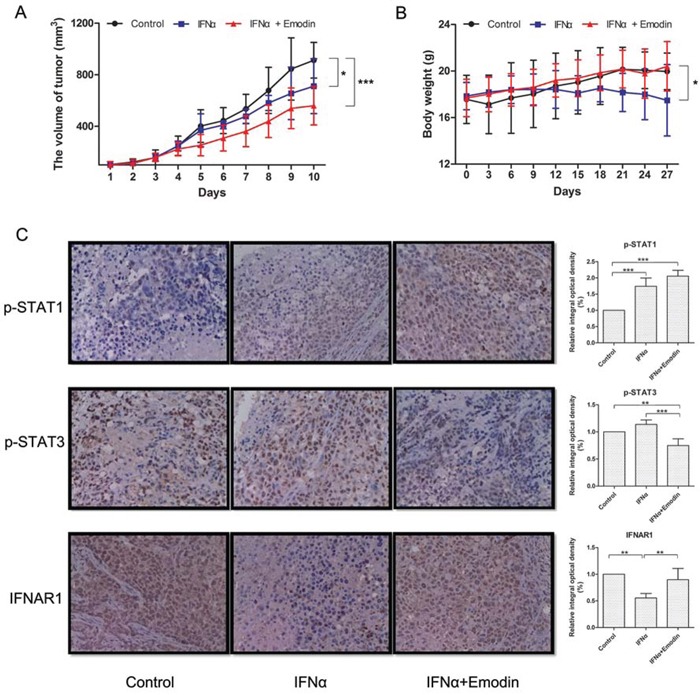
Emodin promotes the antiproliferative effect of IFN-α *in vivo* Huh-7 cells (5 × 10^6^ cells/mice) were injected subcutaneously into the right flank of the mice. The animals were randomly divided into three groups based on tumor volume (about 100 mm^3^). Group I was treated with vehicle, group II was treated with IFN-α and group III was treated with a combination of IFN-α and emodin. Tumor volume (A) and body weight (B) were measured at 3-day intervals. (*) *p* < 0.05, (**) *p* < 0.01, (***) *p* < 0.001 vs. control or IFN-α (*n* = 6). (C) The expressions of p-STAT1, p-STAT3, and IFNAR1 in tumor tissues were measured by immunohistochemistry. Typical images were obtained by Nikon Ti-E microscope (× 400). The relative integral optical density was normalized to control. (**) *p* < 0.01, (***) *p* < 0.001 vs. control or IFN-α (*n* = 6).

To examine whether emodin promotes the antiproliferative effect of IFN-α *in vivo*, we assessed the antiproliferative effect of emodin in combination with IFN-α in nude mice bearing Huh7 human hepatocellular carcinoma cells. As shown in Figure 8A, treatment with IFN-α caused a moderate suppression of tumor volume, with reduction of about 22%. Notably, treatment with IFN-α plus emodin resulted in a synergistic inhibition on tumor growth, with reduction of about 39%. Meanwhile, the body weights of IFN-α plus emodin treated mice were slightly heavier than the body weights of IFN-α treated mice (Figure 8B), which suggested that emodin may decrease the adverse effects of IFN-α on animal. Subsequently, the effects of emodin on IFN-α-mediated JAK/STAT signaling were evaluated in tumor tissues using immunohistochemistry method. As shown in Figure 8C, IFN-α treatment alone upregulated the tyrosine phosphorylation of STAT1 in tumor tissues compared with the control group, and the combination treatment of IFN-α plus emodin further promoted the phosphorylation of STAT1 compared with that of IFN-α treatment alone. Furthermore, high expression of phosphorylated STAT3 was observed in tumor tissues. However, IFN-α treatment increased the phosphorylation level of STAT3, which could be decreased by emodin. In addition, emodin also significantly inhibited the IFN-α-stimulated degradation of IFNAR1. These results suggest that emodin can promote the antiproliferative effect of IFN-α *in vivo*.

## DISCUSSION

Inhibition of proteasome activity has emerged as a new treatment strategy for cancer [21]. This approach was validated by the FDA through its approval of the proteasome inhibitor bortezomib (Velcade, PS314) for the treatment of multiple myeloma and mantle cell lymphoma [22, 23]. However, there are restrictions on the use of bortezomib, including dose limiting toxicity (particularly peripheral neuropathy), limited activity in solid tumors, drug resistance, and the requirement for intravenous administration [24]. These limitations of bortezomib promoted the development of a new generation of structurally distinct proteasome inhibitors [25]. In this work, we conducted an ubiquitin-independent, luciferase-based screening to identify novel proteasome-inhibiting compounds based on the ODC degron, which has been used for green fluorescent protein-based screening of proteasome inhibitors [26]. We found that emodin significantly increased the activity of luciferase-cODC in a manner similar to bortezomib. This result indicates that emodin is a proteasome inhibitor and was further supported by the finding that emodin potently inhibited the 3 peptidase activities of the purified 26S proteasome and increased accumulation of ubiquitinated proteins in cells. Emodin most potently inhibited caspase-like activity (IC_50_ 0.24 μM), followed by chymotrypsin-like activity (IC_50_ 1.22 μM), and finally trypsin-like activity (IC_50_ 20.85 μM). These results are similar to the reported finding that flavones had much stronger inhibitory effects on chymotrypsin-like and caspase-like activities in comparison with their effects on trypsin-like activity [27]. Molecular docking of emodin to the 3 β subunits responsible for the peptidase activities of the proteasome showed that emodin may suppress the caspase-like activity of the β1 subunit mainly through H-bonding and hydrophobic interaction, whereas it may suppress the trypsin-like activity of the β2 subunit and the chymotrypsin-like activity of the β5 subunit mainly through hydrophobic interaction. The amino acids predicted to be affected by emodin are also different from those affected by flavonoids [28]. The chymotrypsin-like sites of the proteasome have long been considered as the only suitable targets for drug development. Bortezomib and all drugs presently undergoing trials (carfilzomib, NPI-0052, CEP-18770, MLN-9708 and ONX-0912) were developed to inhibit chymotrypsin-like activity of 26S proteasome [21]. However, bortezomib, CEP-18770, and MLN-9708 also inhibit the caspase-like activity [29–31], whereas NPI-0052 also inhibits the trypsin-like and caspase-like activities [32]. Therefore, emodin may inhibit the 26S proteasome in a manner similar to bortezomib and represents a new tool that can be used to understand the mechanisms of the 26S proteasome.

Most marketed antiviral drugs directly inhibit viral replication by targeting viral proteins, but such drugs gradually lose their efficacy due to rapid viral mutation [33]. Therefore, the JAK/STAT pathway, a major antiviral defense system in the human body, is an appropriate target for new antiviral and anticancer lead compounds. Despite the discovery of natural inhibitors of the JAK/STAT pathway, activators of this pathway are rarely reported [34]. In this study, we found that emodin activated JAK/STAT signaling and enhanced the antiproliferative effect of IFN-α. Inhibition of IFN-α-stimulated STAT3 activation by emodin is consistent with previous observations that emodin inhibits Jak2 to suppress activation of STAT3 in tumor cells [15, 16]. Activation of STAT1 is also negatively regulated by STAT3 [35]. Therefore, this inhibitory effect of emodin on STAT3 may also explain the promoting effect of emodin on JAK/STAT signaling. Type I IFNs induce IFNAR1 endocytosis and lysosomal degradation, which is regulated by the 26S proteasome [7, 8]. In this study, we found that emodin significantly increased IFNAR1 accumulation, indicating that it may stabilize IFN-bound IFNAR1 via proteasome inhibition. Ubiquitination of IFNAR1 is required for IFNAR1 endocytosis and degradation [7]. Here, we found that IFN-α treatment could increase IFNAR1 ubiquitination, which was significantly inhibited by MG132 or emodin. This result indicated that emodin suppressed IFNAR1 degradation by inhibition of the 26S proteasome, similar to a previous finding that MG132 and lysosomal inhibitors promoted IFNAR1 accumulation [8]. The manner in which inhibition of 26S proteasome activity decreases IFNAR1 ubiquitination is unknown. It is possible that inhibition of the 26S proteasome suppresses the activity of E3 ubiquitin ligases SCF^βTrcp^ and/or SCF^HOB^, which are responsible for IFNAR1 ubiquitination [7, 36], or promotes the activity of an unknown deubiquitinase responsible for decreasing IFNAR1 ubiquitination. After several decades of intense clinical research, the great promise of type I IFNs as anticancer “wonder drugs” has, regrettably, not been fulfilled. The severe side effects and low efficacy of type I IFN-based pharmaceuticals greatly limits use of these drugs and has reduced the enthusiasm of clinical oncologists for type I IFN-based therapeutic modalities. The inefficacy of IFN is postulated to be correlated to activation of numerous signaling pathways that leads to elimination of IFNAR in cancer cells and benign cells that contribute to tumor tissue. Activation of these eliminative pathways enables mitigation of type I IFN-driven suppression of tumorigenesis and elicits the primary refractoriness of tumors to type I IFN-based pharmaceuticals [37]. In this study, we found for the first time that emodin can promote IFNAR1 accumulation by inhibiting the activity of the 26S proteasome and thus potentiate the antiproliferative effect of IFN-α in cancer cells, suggesting that emodin is a potential adjuvant therapeutic that could be used to potentiate the efficacy of type I IFNs in the clinic.

Emodin is capable of inhibiting several inflammatory biomarkers that play crucial roles in the development of inflammatory diseases and cancers [13]. Inhibition of nuclear factor-kappa B (NF-κB), a transcription factor with a central role in the onset of inflammation and tumorigenesis, contributes to the antitumor and anti-inflammatory effects of emodin, but the mechanism underlying this inhibition is unclear [38, 39]. In this study, emodin was identified as an inhibitor of the 26S proteasome, which may explain its inhibitory effects on NF-κB activation and pro-inflammatory cytokine production. NF-κB activation is inhibited by the inhibitor protein I kappa B (IκB), which is degraded through the ubiquitin/26S proteasome pathway [40]. Thus, emodin may also suppress the NF-κB activation by inhibition of 26S proteasome-stimulated IκB degradation, as has been shown for other proteasome inhibitors [40]. The anti-cancer effects of emodin have been studied in tumor cell lines and pre-clinical animal models. Emodin is strong apoptotic agent that induces apoptosis in cancer cell lines by increasing the protein level of p53, a key tumor suppressor involved in inhibition of cellular proliferation [41]. p53 is ubiquitinated by E3 ubiquitin ligase MDM2, which leads to its degradation by the 26S proteasome [42]. Therefore, inhibition of the 26S proteasome by emodin may increase the stability of p53 and thus increase its pro-apoptotic effect, as observed for other proteasome inhibitors [43]. Anthraquinones represent a large family of compounds with diverse biological properties [44]. It will be of interest to examine whether other anthraquinones may exert their biological effects partly through inhibition of the 26S proteasome, as emodin does. Low levels of IFN-α/β are produced even in the absence of viral infection to keep the constitutive weak IFN-α/β and elicit rapid strong cellular responses against infection [45]. Therefore, chemical compounds that promote the effects of type I IFNs may be a new means to enhance their efficacy and reduce side effects. We previously found that luteolin, a natural flavonoid, enhances the antiproliferative effect of IFN-α/β on cancer cells by augmenting the activation of JAK/STAT signaling. Such potentiation is achieved via decreases in intracellular cAMP levels through activation of IFNAR2-bound phosphodiesterase activity and subsequent PKA-stimualted tyrosine phosphatase SHP-2 inhibition [3], which is quite different from the mechanism of emodin reported here. The proteasome also participates in the internalization and degradation of other cell membrane-bound cytokine receptors such as the growth hormone receptor, interleukin-2 receptor, or epidermal growth factor receptor [46–48]. It will be of interest to further investigate whether emodin can stabilize these proteasome-regulated cytokine receptors to exert its anticancer and anti-inflammatory effects.

Taken together, we found that emodin, a natural anthraquinone, is a potent inhibitor of the 26S proteasome. Emodin enhances the antiproliferative effect of IFN-α on cancer cells by promoting the activation of JAK/STAT signaling, which is achieved via inhibition of 26S proteasome-stimulated IFNAR1 degradation. It is of interest to investigate the potential of emodin and other proteasome inhibitors to enhance the efficacy of type I IFNs in the treatment of viral or cancerous diseases.

## MATERIALS AND METHODS

### Reagents

Emodin (purity ≥99%, HPLC-grade) was purchased from Must Biotechnology Co., Ltd. (Chengdu, China). IFN-α (recombinant human IFN-α2a) and IFN-β (recombinant human IFN-β1b) were purchased from ProSpec-Tany Techno Gene Ltd. (Shanghai, China). Bortezomib was purchased from Santa Cruz Biotechnology (Santa Cruz, CA, USA). MG132 was purchased from Sigma-Aldrich (Shanghai, China). The pSV-β-galactosidase expression plasmid was purchased from Promega (Beijing, China).

### Cell culture

Human embryonic kidney 293A (HEK293A) cells (Qbiogene, Carlsbad, CA, USA) and HeLa human cervical cancer cells (American Type Culture Collection, Manassas, VA, USA) were maintained in Dulbecco's modified Eagle's medium (DMEM; Invitrogen, Carlsbad, CA, USA) containing 10% fetal calf serum (Invitrogen) and 1% penicillin/streptomycin at 37°C in a 5% CO_2_ atmosphere. The HepG2-ISRE-Luc2 cell line was established and maintained as previously reported [17].

### Establishment of the HEK293A-luciferase-cODC cell line and screening

To obtain the pCIneo-luciferase-cODC plasmid, the C-terminus (37 amino acids) of mouse ornithine decarboxylase (cODC) was amplified through a plasmid containing full-length mouse ODC (kindly provided by Prof. Philip Coffino, University of California, San Francisco). The cODC fragment was subcloned into the pCIneo-luciferase plasmid, in which luciferase amplified from the pGL4.26 vector (Promega) was cloned into the pCI-neo mammalian expression vector (Promega). The HEK293A-luciferase-cODC stable cell line was generated by transfecting HEK293A cells with the pCIneo-luciferase-cODC plasmid using the Trans-EZ transfection reagent (Sunbio Medical Biotechnology, Shanghai, China) in DMEM medium without antibiotics. After 48 h of transfection, the cells were trypsinized and replated in DMEM with 10% calf serum and 500 μg/mL antibiotic G-418. G-418-resistant clones were selected and expanded in several rounds for 1 month. Cells showing sensitive induction of luciferase activity by proteasome inhibitors bortezomib or MG132 were frozen for further use. On the day of the assay, HEK293A-luciferase-cODC cells were seeded at 1 × 10^4^ cells/well in 96-well plates and incubated overnight in a cell incubator. The cells were treated with the test compounds for 3 h. The luciferase activity of the total cell lysate was measured by a Luciferase Reporter Assay System (Promega).

### Measurement of peptidase activity

Purified human 26S proteasome (0.1 μg) (Enzo, Farmingdale, NY, USA) was incubated with or without different concentrations of emodin in 100 μL assay buffer (50mM Tris-HCl, pH 7.5) and 40 μM fluorogenic peptide substrate Suc-Leu-Leu-Val-Tyr-AMC (Enzo), Ac-Arg-Leu-Arg-AMC (Enzo), or Z-Nle-Pro-Nle-Asp-aminoluciferin (Promega) for 2 hours at 37°C. After the incubation, fluorescence was measured using a Thermo Scientific Varioskan Flash multimode reader.

### Western blotting

Cells were lysed with RIPA buffer supplemented with a protease inhibitor cocktail (Sigma, Shanghai, China). The protein concentration was determined using a BCA protein assay kit (Bestbio, Shanghai, China). Aliquots of total cell lysates (40 μg protein) were mixed with loading buffer, boiled for 5 min, and subjected to 10% SDS-PAGE. Proteins were blotted onto nitrocellulose membranes. The membranes were blocked with 5% bovine serum albumin and then incubated at 4°C overnight with anti-phospho-STAT1 (Abcam, Cambridge, MA, USA), anti-STAT1 (Abcam), anti-phospho-STAT2 (Abcam), anti-STAT2 (Abcam), anti-phospho-STAT3 (SAB Signalway Antibody, College Park, MD), anti-STAT3 (SAB), anti-GAPDH (Abcam), anti-IFNAR1 (Proteintech, Chicago, IL, USA), and anti-ubiquitin (Santa Cruz Biotech) antibodies. Next, the membranes were incubated with a horseradish peroxidase-conjugated secondary antibody (Santa Cruz Biotech) and developed using an enhanced chemiluminescence detection system (Amersham Bioscience, Piscataway, NJ, USA). The intensity of each signal was determined by a computer imaging analysis system (Quantity One, Bio-Rad, Hercules, CA, USA).

### Computational binding simulation

Molecular docking was simulated as previously reported with minor modifications [49]. The crystal structure of the eukaryotic yeast 20S proteasome was obtained from the Protein Database (ref. number 1JD2) and used for all docking studies. The yeast 20S proteasome is structurally very similar to the mammalian 20S proteasome and the active sites are highly conserved between the 2 species [50]. The molecular docking simulation was performed and analyzed using AutoDock 4.2 and AutoDock Vina [51]. AutoDock 4.2, a Lamarkian genetic algorithm method implemented in the program suite, was employed to identify appropriate binding modes and conformation of the ligand molecules. Default parameters (including a distance-dependent dielectric constant) were used as described in the AutoDock manual except as noted below. The docking simulations were performed on a Dell Precision T7600 workstation computer running the Windows 7 Professional operating system. The crystal structure of the 20S proteasome and emodin were prepared for docking by following the default protocols except where noted. The energy-scoring grid was prepared by defining a 20Å × 20Å × 20Å box centered on the N-terminal threonine with a space of 0.2Å between grid points. In the search protocols, the number of genetic runs used was 100 and the number of energy evaluations was set to 5 million. AutoDock reports a docked energy that we have referred to in this article as “docked free energy” because it includes a salvation free energy term. The docked energy also includes the ligand internal energy or the intramolecular interaction energy of the ligand. Docking was chosen by fulfilling the following criteria: proximity to the N-terminal threonine should be 3Å–4Å (a distance suitable for nucleophilic attack) and placement of the A-C ring system of the molecule should be within the S1 hydrophobic pocket. The docked structure of lowest docked free energy was chosen from the orientations/conformations that fit the docking criteria. The probability of adopting the inhibitory conformation was the number of genetic runs (out of 100) in which the molecule docked into the active site and fulfilled the docking criteria. The output from AutoDock and all modeling studies, as well as images, were rendered with PyMOL, which was used to calculate the hydrogen bond distances, as measured between the hydrogen and its assumed binding partner [52].

### Real-time PCR

Total RNA was extracted with TRIzol reagents (Invitrogen) and cDNA was generated using SuperScript III Reverse Transcriptase (Invitrogen) with oligo dT_18_ primer. PKR and 2′5′-OAS1 mRNA was quantified as previously described [3]. The samples were run in triplicate and the relative expression levels of PKR and 2′5′-OAS1 were determined by normalizing the expression of each target to that of GAPDH using the 2^−ΔΔCt^ method.

### Immunoprecipitation

Cell lysates were prepared in ice-cold immunoprecipitation buffer (20 mM Tris-HCl, 200 mM NaCl, 1 mM EDTA, 0.5% Nonidet P-40, pH 8.0) with PMSF and protease cocktail inhibitors. The mixture was centrifuged at 13,000 × *g* for 15 min, cleared with normal immunoglobulin G coupled to agarose beads (protein A/G) for 1 h, incubated overnight at 4°C with anti-IFNAR1 antibodies, and coupled to protein A/G agarose beads for 4 h. The precipitates were washed thrice in PBS buffer, centrifuged at 3,000 × *g* for 3 min, resuspended in 5× loading buffer, and boiled for 5 min. The supernatants were subjected to SDS-PAGE and immunoblotted with anti-ubiquitin antibodies.

### Immunofluorescence staining

HeLa cells were grown in 35mm confocal dish and incubated with cycloheximide (CHX) for 2 h, followed by the addition of emodin for 12 h and treatment with IFN-α (1 × 10^4^ U/mL) for 2 h. Cells were washed with PBS buffer for three times and fixed with 4% paraformaldehyde for 20 min. Then cells were washed with PBS buffer for three times and permeabilized for 5 min with 0.2% Triton X-100 in PBS. Cells were continued to wash with PBS buffer for three times. After blocking with 5% Bovine serum albumin, incubation with antibody IFNAR1 overnight was followed by incubation with secondary antibody with Alexa Fluor 555-Labeled Donkey Anti-Rabbit IgG (Beyotime, shanghai, China) for 1 h. Cells were counterstained with DAPI for 5 min. Cells were imaged by laser scanning confocal microscopy (LeicaMicrosystems TCS-SP8, Wetzlar, Germany).

### Cell viability assay

HeLa cells were seeded in 96-well plates at a density of 5 × 10^3^ cells/well with 100 μL medium. Cultured cells were treated with emodin or a combination of emodin IFN-α at the indicated concentrations. After 72 h, 10 μL Alamar Blue reagent was added to the medium and the cells were incubated for 2–4 h until the color turned from blue to pink. The relative fluorescence intensity was measured using a Thermo Scientific Varioskan Flash multimode reader.

### Colony formation assay

The HeLa cells, at the logarithmic phase, were plated in 6-well plates at the density of 600 cells / well. Cells were treated with emodin in combination with IFN-α and allowed to grow for 12 days to form colonies. A colony was defined as a cluster of more than 50 cells. Then cells were fixed with methanol and stained with 0.1% crystal violet solution for 20 min, and the colonies (> 50 cells) were counted under microscope.

### Animal study

Female 6-week-old athymic nude mice were purchased from Taconic (Oxnard, CA), housed in the Institute of Laboratory Animals, Sichuan Academy of Medical Sciences (Chengdu, China), and fed a normal diet and water ad libitum. All mouse studies were performed according to a protocol approved by the Institute of Laboratory Animals, Sichuan Academy of Medical Sciences, and in line with Guidelines for the welfare and use of animals in cancer research [53]. Briefly, Huh-7 cells (5×10^6^ cells in 200 μl) were suspended in DMEM high glucose medium and injected subcutaneously into the flank of each nude mouse. The length and width of the resulting tumors (in millimeters) were measured every three days with calipers, and then the tumor volume (0.5 × length × width^2^) was calculated. When the tumor volume reached around 100 mm^3^, the mice were randomly divided into the following three groups: one group received intraperitoneal injections of vehicle (olive oil, 0.2 ml/10g, every day), the second group received intraperitoneal injections of interferon α2a (5×10^6^ U/kg, every 3 days) and the third group were intraperitoneal injected with both interferon α2a and emodin (25 mg/kg, every day). Animals were sacrificed by cervical dislocation at day 28 after first therapeutic dose injection and the tumors were resected and fixed with 10% neutral-buffered formalin. They were cut into 5-μm sections after embedding in paraffin. Tumor sections were determined by immunohistochemistry.

### Immunohistochemistry

Tumor tissues were fixed in 10% formalin and embedded in paraffin wax. Five-micrometer sections were cut from the paraffin blocks for immunohistochemical analysis. The paraffin sections were dewaxed with xylene, and hydrated with gradient ethanol. Then, sections were treated in a microwave oven at low power for 10 min in 0.01 M sodium citrate buffer (pH = 6.0) and blocked with 10% Goat serum at room temperature for 2 h. Subsequently, the sections were stained with anti-pSTAT1 (1:100, SAB), anti-pSTAT3 (1:100, SAB), and anti-IFNAR1 (1:100, Zen Bioscience, Chengdu, China) polyclonal rabbit antibodies at 4°C overnight. Sections were then washed with TBST, and endogenous peroxidase was inactivated using 3% hydrogen peroxide for 15 min. Next, sections were incubated with the horseradish peroxidase-labeled secondary antibody (1:1000, Zen Bioscience) for 1 h at room temperature, and antibody-binding sites were visualized by DAB kit (Zhongshan Golden Bridge Biotechnology, Beijing, China). Thereafter, sections were stained with haematine for 10 min, dehydrated and clear by gradient ethanol and xylene, respectively. Finally, the samples were observed under light microscope after sealed with neutral balsam on slides.

### Statistical analysis

Statistical analyses were performed with GraphPad Prism 5.0 software (GraphPad, La Jolla, CA, USA). All experiments were repeated at least thrice and representative results are presented. The data were compared by one-way ANOVA followed by Dunnett's post-hoc test. The differences were considered statistically significant when *p* < 0.05.

## SUPPLEMENTARY MATERIALS AND METHODS FIGURES



## References

[1] Borden EC, Sen GC, Uze G, Silverman RH, Ransohoff RM, Foster GR, Stark GR (2007). Interferons at age 50: past, current and future impact on biomedicine. Nat Rev Drug Discov.

[2] Jonasch E, Haluska FG (2001). Interferon in oncological practice: review of interferon biology, clinical applications, and toxicities. Oncologist.

[3] Tai Z, Lin Y, He Y, Huang J, Guo J, Yang L, Zhang G, Wang F (2014). Luteolin sensitizes the antiproliferative effect of interferon α/β by activation of Janus kinase/signal transducer and activator of transcription pathway signaling through protein kinase A-mediated inhibition of protein tyrosine phosphatase SHP-2 in cancer cells. Cell Signal.

[4] Lucas-Hourani M, Dauzonne D, Jorda P, Cousin G, Lupan A, Helynck O, Caignard G, Janvier G, André-Leroux G, Khiar S, Escriou N, Desprès P, Jacob Y (2013). Inhibition of pyrimidine biosynthesis pathway suppresses viral growth through innate immunity. PLoS Pathog.

[5] Konishi H, Okamoto K, Ohmori Y, Yoshino H, Ohmori H, Ashihara M, Hirata Y, Ohta A, Sakamoto H, Hada N, Katsume A, Kohara M, Morikawa K (2012). An orally available, small-molecule interferon inhibits viral replication. Sci Rep.

[6] van Boxel-Dezaire AH, Rani MR, Stark GR (2006). Complex modulation of cell type-specific signaling in response to type I interferons. Immunity.

[7] Kumar KG, Krolewski JJ, Fuchs SY (2004). Phosphorylation and specific ubiquitin acceptor sites are required for ubiquitination and degradation of the IFNAR1 subunit of type I interferon receptor. J Biol Chem.

[8] Ragimbeau J, Dondi E, Alcover A, Eid P, Uzé G, Pellegrini S (2003). The tyrosine kinase Tyk2 controls IFNAR1 cell surface expression. EMBO J.

[9] Soond SM, Townsend PA, Barry SP, Knight RA, Latchman DS, Stephanou A (2008). ERK and the F-box protein βTRCP target STAT1 for degradation. J Biol Chem.

[10] Didcock L, Young DF, Goodbourn S, Randall RE (1999). The V protein of simian virus 5 inhibits interferon signalling by targeting STAT1 for proteasome-mediated degradation. J Virol.

[11] Lee Y, Hyung SW, Jung HJ, Kim HJ, Staerk J, Constantinescu SN, Chang EJ, Lee ZH, Lee SW, Kim HH (2008). The ubiquitin-mediated degradation of Jak1 modulates osteoclastogenesis by limiting interferon-β-induced inhibitory signaling. Blood.

[12] Srinivas G, Babykutty S, Sathiadevan PP, Srinivas P (2007). Molecular mechanism of emodin action: transition from laxative ingredient to an antitumor agent. Med Res Rev.

[13] Shrimali D, Shanmugam MK, Kumar AP, Zhang J, Tan BK, Ahn KS, Sethi G (2013). Targeted abrogation of diverse signal transduction cascades by emodin for the treatment of inflammatory disorders and cancer. Cancer Lett.

[14] Zheng Y, Qin H, Frank SJ, Deng L, Litchfield DW, Tefferi A, Pardanani A, Lin FT, Li J, Sha B, Benveniste EN (2011). A CK2-dependent mechanism for activation of the JAK-STAT signaling pathway. Blood.

[15] Muto A, Hori M, Sasaki Y, Saitoh A, Yasuda I, Maekawa T, Uchida T, Asakura K, Nakazato T, Kaneda T, Kizaki M, Ikeda Y, Yoshida T (2007). Emodin has a cytotoxic activity against human multiple myeloma as a Janus-activated kinase 2 inhibitor. Mol Cancer Ther.

[16] Subramaniam A, Shanmugam MK, Ong TH, Li F, Perumal E, Chen L, Vali S, Abbasi T, Kapoor S, Ahn KS, Kumar AP, Hui KM, Sethi G (2013). Emodin inhibits growth and induces apoptosis in an orthotopic hepatocellular carcinoma model by blocking activation of STAT3. Br J Pharmacol.

[17] Tai ZF, Zhang GL, Wang F (2012). Identification of small molecule activators of the janus kinase/signal transducer and activator of transcription pathway using a cell-based screen. Biol Pharm Bull.

[18] Zhang M, Pickart CM, Coffino P (2003). Determinants of proteasome recognition of ornithine decarboxylase, a ubiquitin-independent substrate. EMBO J.

[19] Erales J, Coffino P (2014). Ubiquitin-independent proteasomal degradation. Biochim Biophys Acta.

[20] Hoyt MA, Zhang M, Coffino P (2005). Probing the ubiquitin/proteasome system with ornithine decarboxylase, a ubiquitin-independent substrate. Methods Enzymol.

[21] Adams J (2004). The proteasome: a suitable antineoplastic target. Nat Rev Cancer.

[22] Richardson PG, Sonneveld P, Schuster MW, Irwin D, Stadtmauer EA, Facon T, Harousseau J, Ben-Yehuda D, Lonial S, Goldschmidt H, Reece D, San-Miguel JF (2005). Bortezomib or high-dose dexamethasone for relapsed multiple myeloma. N Engl J Med.

[23] Kane RC, Dagher R, Farrell A, Ko CW, Sridhara R, Justice R, Pazdur R (2007). Bortezomib for the treatment of mantle cell lymphoma. Clin Cancer Res.

[24] Richardson PG, Hideshima T, Anderson KC (2003). Bortezomib (PS-341): a novel, first-in-class proteasome inhibitor for the treatment of multiple myeloma and other cancers. Cancer Control.

[25] Kisselev AF, van der Linden WA, Overkleeft HS (2012). Proteasome inhibitors: an expanding army attacking a unique target. Chem Biol.

[26] Rickardson L1, Wickström M, Larsson R, Lövborg H (2007). Image-based screening for the identification of novel proteasome inhibitors. J Biomol Screen.

[27] Chang TL (2009). Inhibitory effect of flavonoids on 26S proteasome activity. J Agric Food Chem.

[28] Mozzicafreddo M, Cuccioloni M, Cecarini V, Eleuteri AM, Angeletti M (2009). Homology modeling and docking analysis of the interaction between polyphenols and mammalian 20S proteasomes. J Chem Inf Model.

[29] Altun M, Galardy PJ, Shringarpure R, Hideshima T, LeBlanc R, Anderson KC, Ploegh HL, Kessler BM (2005). Effects of PS-341 on the activity and composition of proteasomes in multiple myeloma cells. Cancer Res.

[30] Kupperman E, Lee EC, Cao Y, Bannerman B, Fitzgerald M, Berger A, Yu J, Yang Y, Hales P, Bruzzese F, Liu J, Blank J, Garcia K (2010). Evaluation of the proteasome inhibitor MLN9708 in preclinical models of human cancer. Cancer Res.

[31] Piva R, Ruggeri B, Williams M, Costa G, Tamagno I, Ferrero D, Giai V, Coscia M, Peola S, Massaia M, Pezzoni G, Allievi C, Pescalli N (2008). CEP-18770: A novel, orally active proteasome inhibitor with a tumor-selective pharmacologic profile competitive with bortezomib. Blood.

[32] Chauhan D, Catley L, Li G, Podar K, Hideshima T, Velankar M, Mitsiades C, Mitsiades N, Yasui H, Letai A, Ovaa H, Berkers C, Nicholson B (2005). A novel orally active proteasome inhibitor induces apoptosis in multiple myeloma cells with mechanisms distinct from bortezomib. Cancer Cell.

[33] Richman DD (2006). Antiviral drug resistance. Antiviral Res.

[34] Lin Y, Wang F, Zhang GL (2014). Natural products and their derivatives regulating the janus kinase/signal transducer and activator of transcription pathway. J Asian Nat Prod Res.

[35] Regis G, Pensa S, Boselli D, Novelli F, Poli V (2008). Ups and downs: the STAT1:STAT3 seesaw of Interferon and gp130 receptor signalling. Semin Cell Dev Biol.

[36] Kumar KG, Tang W, Ravindranath AK, Clark WA, Croze E, Fuchs SY (2003). SCF^HOS^ ubiquitin ligase mediates the ligand-induced down-regulation of the interferon-α receptor. EMBO J.

[37] Fuchs SY (2013). Hope and fear for interferon: the receptor-centric outlook on the future of interferon therapy. J Interferon Cytokine Res.

[38] Huang Q, Shen HM, Ong CN (2004). Inhibitory effect of emodin on tumor invasion through suppression of activator protein-1 and nuclear factor-κB. Biochem Pharmacol.

[39] Li HL, Chen HL, Li H, Zhang KL, Chen XY, Wang XW, Kong QY, Liu J (2005). Regulatory effects of emodin on NF-κB activation and inflammatory cytokine expression in RAW 264. 7 macrophages. Int J Mol Med.

[40] Alkalay I, Yaron A, Hatzubai A, Orian A, Ciechanover A, Ben-Neriah Y (1995). Stimulation-dependent IκBα phosphorylation marks the NF-κB inhibitor for degradation via the ubiquitin-proteasome pathway. Proc Natl Acad Sci USA.

[41] Shieh DE, Chen YY, Yen MH, Chiang LC, Lin CC (2004). Emodin-induced apoptosis through p53-dependent pathway in human hepatoma cells. Life Sci.

[42] Haupt Y, Maya R, Kazaz A, Oren M (1997). Mdm2 promotes the rapid degradation of p53. Nature.

[43] Li M1, Brooks CL, Wu-Baer F, Chen D, Baer R, Gu W (2003). Mono-versus polyubiquitination: differential control of p53 fate by Mdm2. Science.

[44] Huang Q, Lu G, Shen HM, Chung MC, Ong CN (2007). Anti-cancer properties of anthraquinones from rhubarb. Med Res Rev.

[45] Taniguchi T, Takaoka A (2001). A weak signal for strong responses: Interferon-α/β revisited. Nat Rev Mol Cell Bio.

[46] Longva KE, Blystad FD, Stang E, Larsen AM, Johannessen LE, Madshus IH (2002). Ubiquitination and proteasomal activity is required for transport of the EGF receptor to inner membranes of multivesicular bodies. J Cell Biol.

[47] Yu A, Malek TR (2001). The proteasome regulates receptor-mediated endocytosis of interleukin-2. J Biol Chem.

[48] van Kerkhof P, Govers R, Alves dos Santos CM, Strous GJ (2000). Endocytosis and degradation of the growth hormone receptor are proteasome-dependent. J Biol Chem.

[49] Chen D, Daniel KG, Chen MS, Kuhn DJ, Landis-Piwowar KR, Dou QP (2005). Dietary flavonoids as proteasome inhibitors and apoptosis inducers in human leukemia cells. Biochem Pharmacol.

[50] Groll M, Ditzel L, Löwe J, Stock D, Bochtler M, Bartunik HD, Huber R (1997). Structure of 20S proteasome from yeast at 2.4Å resolution. Nature.

[51] Trott O, Olson AJ (2010). AutoDock Vina: Improving the speed and accuracy of docking with a new scoring function, efficient optimization, and multithreading. J Comput Chem.

[52] Vetrivel U, Pilla K (2008). Open discovery: An integrated live Linux platform of Bioinformatics tools. Bioinformation.

[53] Workman P, Aboagye EO, Balkwill F, Balmain A, Bruder G, Chaplin DJ, Double JA, Everitt J, Farningham DAH, Glennie MJ, Kelland LR, Robinson V, Stratford IJ, Tozer GM, Watson S, Wedge SR, Eccles SA (2010). Guidelines for the welfare and use of animals in cancer research. Br J Cancer.

