# Supplementary material for: Emodin potentiates the antiproliferative effect of interferon α/β by activation of JAK/STAT pathway signaling through inhibition of the 26S proteasome
Source: Oncotarget. 2015 Dec 14;7(4):4664–79. doi: 10.18632/oncotarget.6616 (PMC4826234; doi:10.18632/oncotarget.6616)
Supplement: Supplementary file 1 [file oncotarget-07-4664-s001.pdf]

MATERIALS AND METHODS

Reagent

The Jak inhibitor ruxolitinib was purchased from Selleck (Shanghai, China).

Plasmid construction and proteasome activity assay in vivo.

The pEGFP-CL1 plasmid was obtained by inserting the CL1 sequence (gcttgtaagaactggttctcttctttgtctcacttcgttatccacttgtga) through EcoRI and BamHI endonuclease restriction sites to the pEGFP-C2 vector (CLONTECH, Palo Alto, CA). HeLa cells were seeded in 6-well plates and transiently transfected with 2.5 μg pEGFP-CL1 plasmid using Lipofectamine™ 2000 Transfection Reagent (Invitrogen) in DMEM medium without antibiotics. After 4–6 h, the transfection medium (OPTI-MEM, Gibco-Invitrogen) was replaced with the normal medium (DMEM with 10% FBS). After 24 h of transfection, cells were treated with MG132 or emodin for 6 h. The CL1 degradation was shown by GFP expression level. The images was obtained by fluorescence microscopy. The pEGFP-C2 plasmid lacking CL1 sequence was used as a positive control.

Knockdown of STAT1 expression by siRNA transfection

The siRNA (5'-GGAUAAUUUUCAGGAAGAC dTdT-3') to target human STAT1 and negative control siRNA (mock-siRNA) were synthesized by RiboBio Co., Ltd. (Guangzhou,China). Transient transfection of HeLa cells with siRNA (100 nm) was performed using Lipofectamine™ 2000 Transfection Reagent (Invitrogen) according to the manufacturer's protocol. For western blot experiments, cells were plated on 6-well dishes and cultured for 48 h after transfection to 100% confluence. For anti-proliferative studies, cells were plated on 24-well plates and transfected with siRNA for 48 h, followed by the treatment with emodin (10 μM) and IFN-α (1 × 104 U/mL) for another 72 h.

SUPPLEMENTARY FIGURES

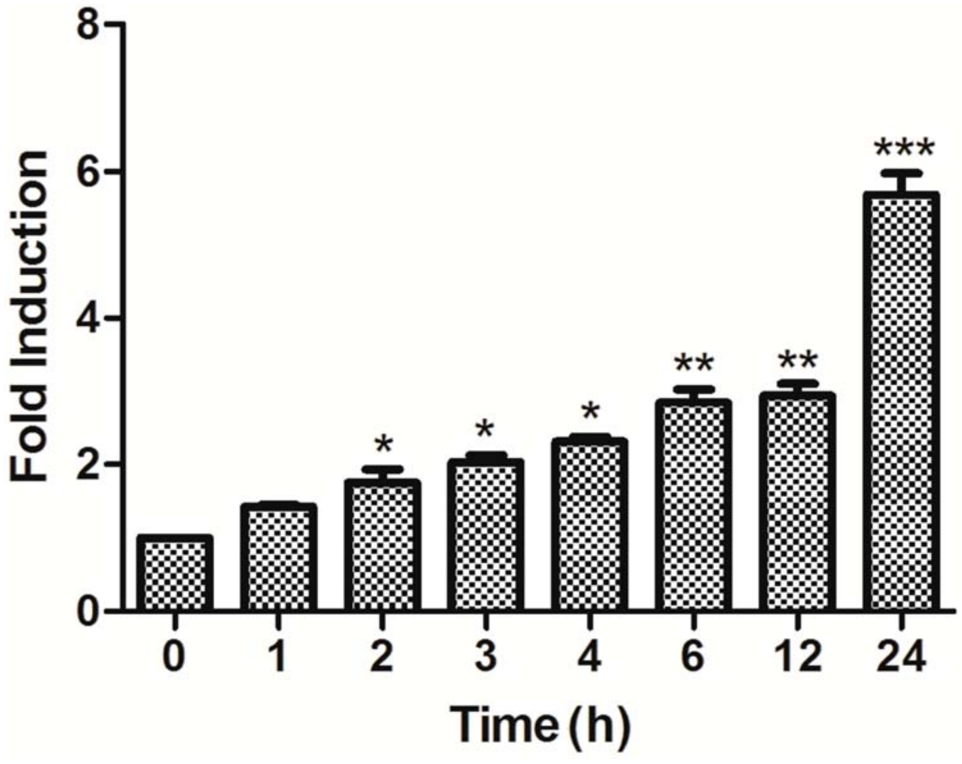

Supplementary Figure S1: Effect of MG132 on luciferase-cODC expression. HEK293A-luciferase-cODC cells were seeded into a 96-well plate and treated with MG132 (10 μM) for the indicated time. Cell lysates were measured for luciferase activity. (*) $p < 0.05$, (**) $p < 0.01$, (***) $p < 0.001$ compared to the DMSO control.

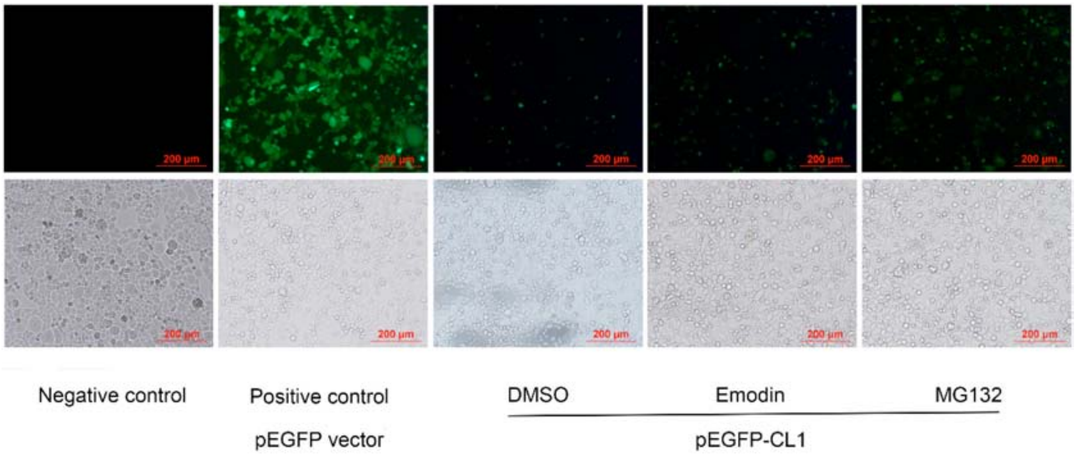

Supplementary Figure S2: Emodin inhibits the activity of the 26S proteasome in vivo. HeLa cells grown in 6-well plates were transiently transfected with GFP-CL1 or GFP for 24 h, followed by the treatment with MG132 (20 μM) or emodin (20 μM) for 6 h. The GFP expression level was observed by fluorescence microscopy.

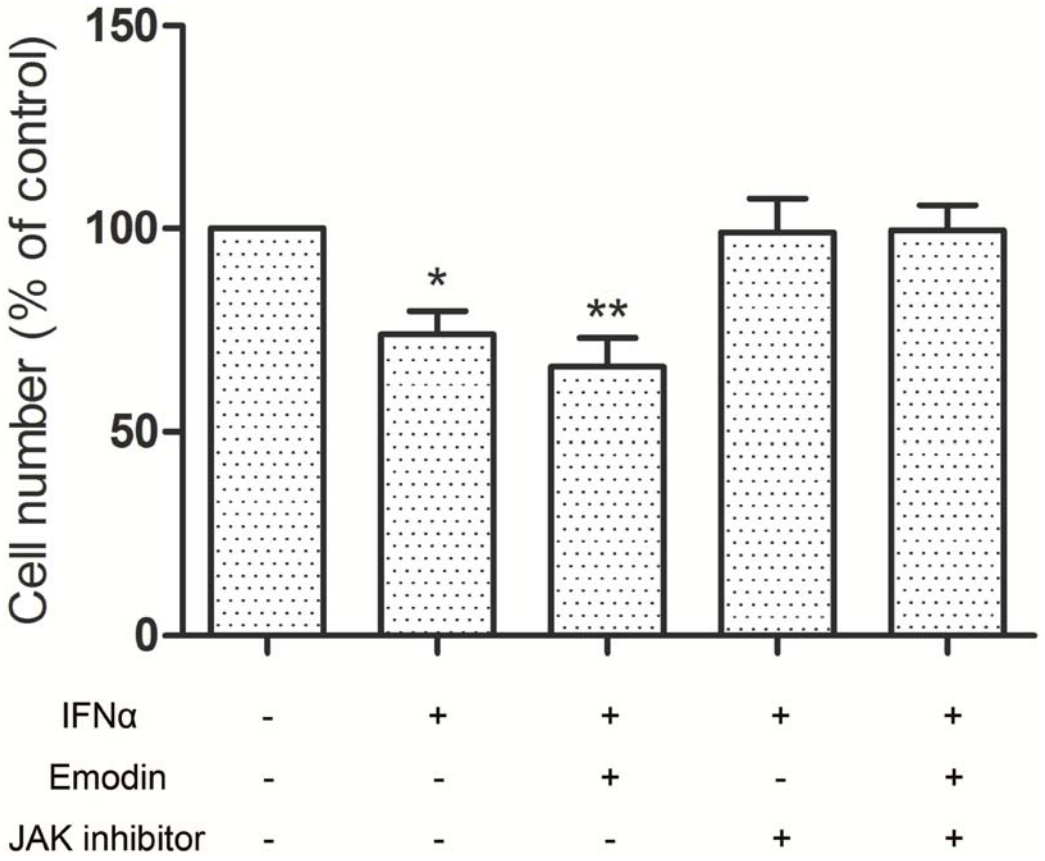

Supplementary Figure S3: Effect of JAK inhibitor on the proliferation of cancer cells. HeLa cells were seeded in 96-well plates and incubated with JAK inhibitor (Ruxolitinib, 1 μM) for 1 h, followed by the addition of emodin (10 μM) for 2 h and the treatment with IFN-α (1 × 10^4 U/mL) for 72 h. The numbers of cells were recorded by a counting board. (*) $p < 0.05$, (**) $p < 0.01$, (***) $p < 0.001$ vs. control ($n = 3$).

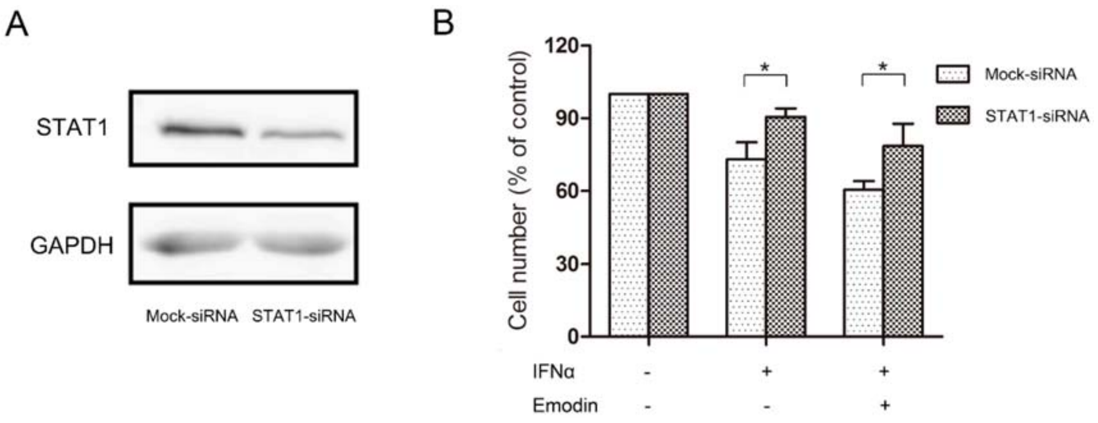

Supplementary Figure S4: Effect of STAT1 knockdown on the proliferation of cancer cells. A. HeLa cells cultured in 6-well plates were transfected with Mock-siRNA or STAT1-siRNA for 48 h. The cell lysates were immunoblotted with STAT1 antibody and GAPDH staining was shown as a loading control. **B.** HeLa cells were seeded in 24-well plates. After transfection with Mock-siRNA or STAT1-siRNA for 48 h, cells were incubated with 10 μM emodin for 2 h and treated with IFN-α (1×10^4 U/mL) for another 72 h. The numbers of cells were recorded by a counting board. (*) $p < 0.05$, (**) $p < 0.01$, (***) $p < 0.001$ vs. Mock-siRNA control ($n = 3$).
